# Color-coded parametric imaging support display of vessel hemorrhage—an *in vitro* experiment and clinical validation study

**DOI:** 10.3389/fcvm.2024.1387421

**Published:** 2024-06-20

**Authors:** Yi Chen, Wenji Xu, Jiaxin Liu, Chao Zhao, Xiaojing Cao, Rong Wang, Duiping Feng, Ruiping Zhang, Xiang Zhou

**Affiliations:** ^1^ Shanxi Bethune Hospital, Shanxi Academy of Medical Sciences, Tongji Shanxi Hospital, Third Hospital of Shanxi Medical University, Taiyuan, China; ^2^ Tongji Hospital, Tongji Medical College, Huazhong University of Science and Technology, Wuhan, China; ^3^ Department of Oncology and Vascular Intervention, First Hospital of Shanxi Medical University, Taiyuan, Shanxi, China; ^4^ Shanxi Provincial Clinical Research Center for Interventional Medicine (202204010501004), Taiyuan, China; ^5^ College of Medical Imaging, Shanxi Medical University, Taiyuan, Shanxi, China; ^6^ College of Electronic Information Engineering, Beihang University, Beijing, China; ^7^ Department of Interventional Therapy, National Cancer Center/National Clinical Research Center for Cancer/Cancer Hospital, Chinese Academy of Medical Sciences and Peking Union Medical College, Beijing, China; ^8^ The Radiology Department of Shanxi Provincial People's Hospital, The Fifth Hospital of Shanxi Medical University, Taiyuan, China

**Keywords:** active bleeding, DSA, color-coded parametric imaging, time of arrival, diagnosis

## Abstract

**Background:**

Digital Subtraction Angiography (DSA) is currently the most effective diagnostic method for vascular diseases, but it is still subject to various factors, resulting in uncertain diagnosis. Therefore, a new technology is needed to help clinical doctors improve diagnostic accuracy and efficiency.

**Purpose:**

The objective of the study was to investigate the effect of utilizing color-coded parametric imaging techniques on the accuracy of identifying active bleeding through DSA, the widely accepted standard for diagnosing vascular disorders.

**Methods:**

Several variables can delay the diagnosis and treatment of active bleeding with DSA. To resolve this, we carried out an *in vitro* simulation experiment to simulate vascular hemorrhage and utilized five color-coded parameters (area under curve, time to peak, time-of-arrival, transit time, and flow rate of contrast agent) to determine the optimal color coding parameters. We then verified it in a clinical study.

**Results:**

Five different color-coded parametric imaging methods were compared and the time-of-arrival color coding was the most efficient technique for diagnosing active hemorrhage, with a statistically significant advantage (*P *< 0.001). In clinical study, 135 patients (101 with confirmed bleeding and 34 with confirmed no bleeding) were collected. For patients whose bleeding could not be determined using DSA alone (55/101) and whose no bleeding could not be diagnosed by DSA alone (35/55), the combination of time-of-arrival color parametric imaging was helpful for diagnosis, with a statistically significant difference (*P *< 0.01 and *P *= 0.01).

**Conclusions:**

The time-of-arrival color coding imaging method is a valuable tool for detecting active bleeding. When combined with DSA, it improves the visual representation of active hemorrhage and improves the efficiency of diagnosis.

## 1 Introduction

Digital Subtraction Angiography (DSA) is currently the most reliable method for diagnosing and evaluating vascular lesions, especially hemorrhagic lesions (1, 2). It allows for accurate treatment and evaluation of therapeutic efficacy, making it the gold standard for vascular disease (3–5). However, the diagnosis of bleeding through DSA images often relies on subjective judgment by radiologists with specialized training and experience, which can be affected by external factors such as the expertise of the physician, image quality, and equipment. There are several reasons why bleeding points may not be clearly visible on DSA images: poor image quality due to factors such as breathing, intestinal gas, bleeding site, and instrument settings; the presence of other abnormal vessels that obscure the bleeding point, such as those found in tumors; overlapping, tortuous, leaky, or irregular vessels that make the bleeding point hard to distinguish; and slender bleeding vessels. These factors can hinder accurate diagnosis of bleeding and may lead to missed or incorrect diagnoses, missing the optimal treatment time (6). Therefore, it is important to develop image technologies that can assist physicians in identifying bleeding quickly and accurately.

Computer-Aided Diagnosis (CAD) technology, which combines computer graphics and image processing, has been shown to improve diagnostic accuracy and ease of diagnosis (7). Color-coded parametric imaging is a part of CAD that converts grayscale images to color-coded images using computer algorithms. It has been used in the diagnosis and treatment of cerebrovascular diseases and has been found to be highly beneficial in the diagnosis and treatment of these conditions (8, 9). Converting grayscale images to color images may be particularly useful for displaying hemorrhagic lesions (10–13). However, there is limited research on the use of color-coded parametric imaging in the diagnosis of hemorrhagic diseases. In this study, we conducted an *in vitro* simulation experiment using DSA to diagnose hemorrhagic diseases and compared the ability of five different color-coded imagings to display bleeding lesions. Our goal was to identify the optimal color-coded imaging technique and provide a new approach for displaying bleeding lesions in the diagnosis of hemorrhagic diseases.

## 2 Materials and methods

### 2.1 Design of active bleeding simulation experiments

The purpose of this *ex-vivo* study was to use the fibers of a hemodialysis device to simulate small blood vessels in the human body and to simulate bleeding by cutting the fibers of the dialyzer. The parameters of the dialyzer and the experimental protocols are provided in the Supplementary Material S1.

### 2.2 Orthogonal experimental design for DSA imaging parameter optimization

In this study, we initially selected 8 parameters that may affect the performance of DSA images (14, 15): (1) the speed of contrast agent injection, (2) the timing of contrast agent injection, (3) the dose of x-ray, (4) the DSA imaging frame rate, (5) the pressure of contrast agent injection, (6) the flow velocity, 6) flow rate, (7) number of broken fibers (the number of cutting fibers of the dialyzer to simulate bleeding) and (8) the distance of contrast agent injection from the injection point to the dialyzer. In order to obtain a sufficient number of experiments, we used an orthogonal experimental design to create 27 experimental groups (Supplementary Material S2).

### 2.3 Research on methods and mathematical models of color-coded parametric imaging

In this study, we employed color-coded parametric imaging as method to transform 2D DSA raw data into color images. This process was divided into the following 5 steps.

Step 1: Image enhancement. This step aims to reduce noise in the image and preserve important information for further analysis. In our study, we utilized a Gaussian low-pass filter for noise reduction.

Step 2: Vasculature segmentation. Segmenting blood vessels is a vital process in color-coded parametric imaging, but there is no widely accepted and effective method for doing so. While there are numerous techniques available, in this study, we segmented the vascular region by identifying differences in shape and color compared to the background, which proved to be a successful approach.

Step 3: Time-intensity curve fitting. To analyze the time-intensity relationship of each pixel, we used the bolus curve model to fit the time-intensity curve (16). In this study, a bolus curve model was utilized to compute the time-intensity curve for each pixel in the blood vessel region of a 2D DSA image sequence: $I(t)\;=\;a_{0}+a_{1}\frac{e^{-a_{2}t}}{1+e^{-a_{3}(t-t_{0})}}$. The time-intensity curve is defined by the following formula: *t*_0_ represents the transition point of the perfusion process, *a*_0_ represents the baseline intensity of the contrast agent (i.e., the intensity before the contrast agent arrives), and *a*_1_ represents the intensity increment of the contrast agent (i.e., the increase or decrease of the contrast intensity). *a*_2_ indicates the speed at which the peak intensity of the contrast agent is halved, and *a*_3_ describes the rising slope of the contrast agent intensity (representing the speed of perfusion). The parameters *t*_0_ and *a*_0_ are determined by measuring the amplitude of the contrast agent intensity value change between the current frame and the previous frame of a single pixel in the DSA image sequence. The values of *a*_1_, *a*_2_, and *a*_3_ are calculated using the least square method based on the intensity values of each pixel in the blood vessel area. Once the time-intensity curve is obtained, the parameter data is calculated according to the selected time region. The parameter data includes: (1) AUC: the area under the curve of the time-intensity curve vs. time integration; (2) time to peak: the time from the injection of the contrast agent to the maximum intensity value of a single pixel point; (3) arrival time: the time from the injection of the contrast agent to the first large increase in the gray value of a single pixel (the increase is greater than 15%); (4) pass through time: the time from when the gray value of a single pixel first increases significantly (the increase is greater than 15%) to when it decreases significantly (the decrease is greater than 15%), the subtraction of the two is the transit time; (5) contrast agent flow rate: the speed of contrast agent flow. The calculated parameter value for each pixel determined its gray value, and the corresponding gray image was obtained.

Step 4: Image Pseudo-coloring. We used a grayscale-color transformation method to convert the grayscale images of the five parameters into corresponding color-coded images.

Step 5: Following the pseudo-color processing of the image, the resulting color-coded parametric images are presented on a computer screen for examination. These vivid images depict various perfusion parameters of the blood vessels and can be utilized for a thorough analysis of blood flow dynamics. The diverse colors employed for pseudo-coloring signify distinct values of the parameters, such as AUC, transit time, peak time, arrival time, and contrast agent flow rate. This visual representation enables a swift and intuitive comprehension of the blood flow dynamics within the image.

### 2.4 Color-coded parameter screening experiments

Observers analyzed 27 sets of DSA sequence dynamic images and color parametric images of *ex-vivo* experiments. All observers were physicians with extensive experience in diagnosing DSA (more than 8 years of experience in DSA diagnosis, a total of 3) and were blinded to whether there was breakpoint or not. The process of viewing the images was as follows.

First, the observers watched the DSA sequence images and then answered the following question:

Whether breakpoint can be observed in the DSA sequence images, scored as follows: 1 point: definitely no breakpoint, 2 points: probably no breakpoint, 3 points: uncertain, 4 points: possible breakpoint, 5 points: definitely breakpoint.

The observers then observed the imaging of 5 color-coded parameters and answered the following questions: (1) Whether breakpoint can be observed in the color-coded parametric imaging and scored; (2) Whether breakpoint can be observed in the combination of color-coded parametric imaging and DSA images, and scored.

After the evaluation was completed, if the results of the observers in the groups were inconsistent, three observers were required to discuss and give a consistent answer. Paired Wilcoxon signed-rank tests were used to compare the ability of 5 parameters (AUC, time to peak, arrival time, transit time, contrast agent flow rate) to identify breakpoint. The optimal color parametric imaging for identifying breakpoint was obtained. The Wilcoxon signed-rank test was also applied to compare the capability of identifying breakpoints between DSA images alone and DSA images with color coding parameters added. All statistical procedures were performed using SPSS software (IBM SPSS Statistics version: 23.0), and *P *< 0.05 was considered statistically significant.

### 2.5 Clinical validation study

To validate the results of *ex-vivo* experiments, patients diagnosed with active hemorrhage and those without hemorrhage between January 2019 and June 2022 were recruited from three hospitals:****. All participants underwent DSA. Hemorrhage was diagnosed based on clinical criteria, such as a significant decrease in hemoglobin levels (more than 10 g/L) and stabilization of hemoglobin levels after embolization. Patients without relevant clinical symptoms and undergoing angiography for other treatments were considered as not having hemorrhage.

All patients were divided into two groups: the bleeding group and the non-bleeding group. Three clinicians with at least 8 years of experience in DSA diagnosis reviewed and scored DSA images and color-coded parameter images. The observers were blinded to whether the patients were bleeding or not. The process of viewing the DSA and color-coded parameter images is consistent with *in vitro* experiments, and the scoring criteria are also consistent with *in vitro* experiments. Please refer to Section 2.4 for details.

**Figure 1:**
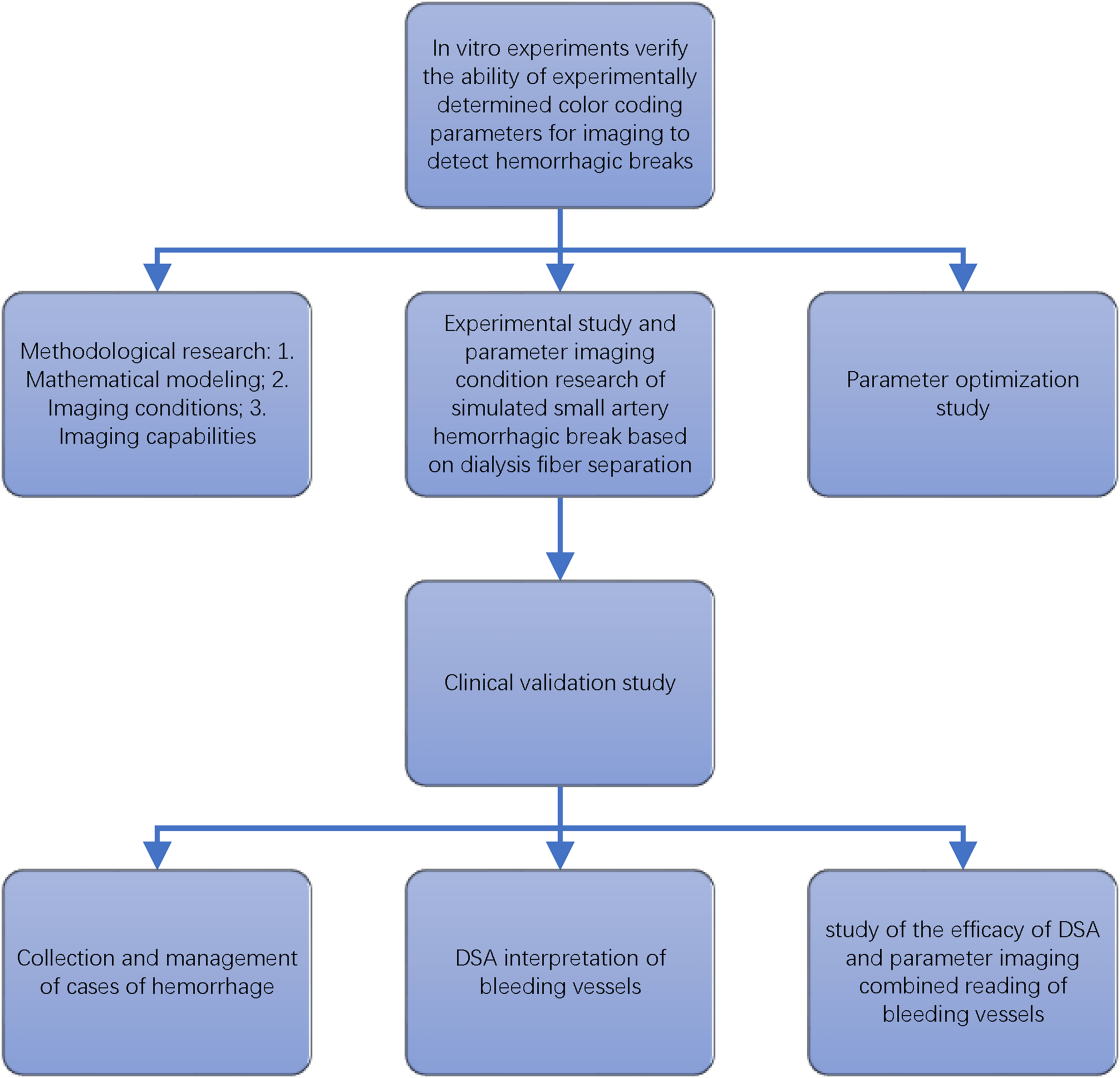
The flow chart of study of color coded parameter imaging.

If the results of the observers in the groups were inconsistent, a consensus was reached after a discussion among the three observers. The Wilcoxon signed-rank test was used to compare the ability of identifying bleeding between simple DSA images and combining color coding parameter imaging for both the bleeding group and the non-bleeding group. The overall experimental process is illustrated in Figure 1.

## 3 Results

### 3.1 Five types of color-coded parametric imaging for identifying breakpoints of fibers

**Figure 2:**
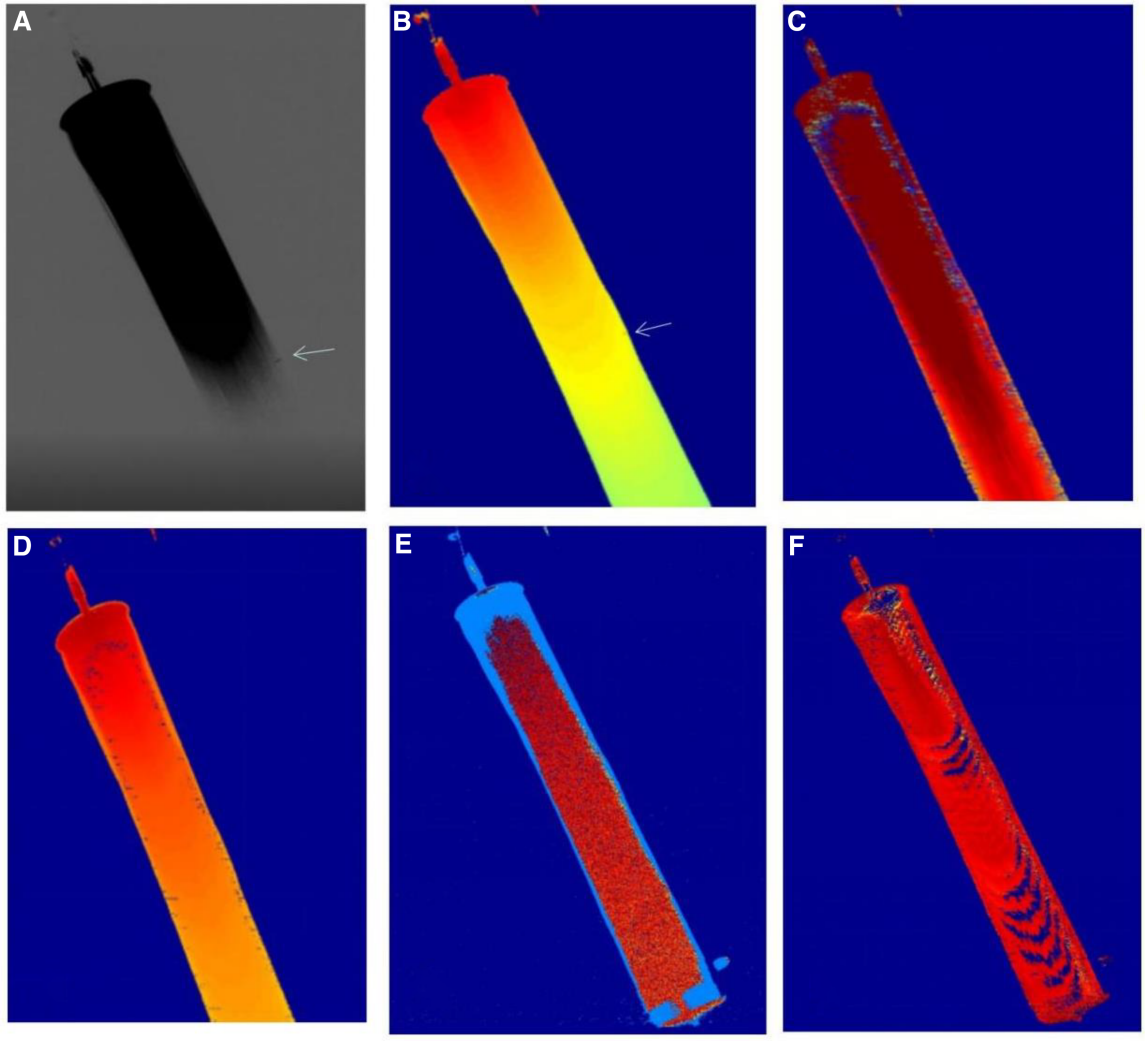
Shows the imaging results of five color parameters of the same DSA image. Figure 1(**A**) shows that DSA can display bleeding, and the time-of-arrival color parameter imaging can also display bleeding Figure 1(**B**), and other color parameters (**C–F**) (AUC, time to peak, transit time, contrast flow rate) were not well visualized for bleeding.

**Figure 3:**
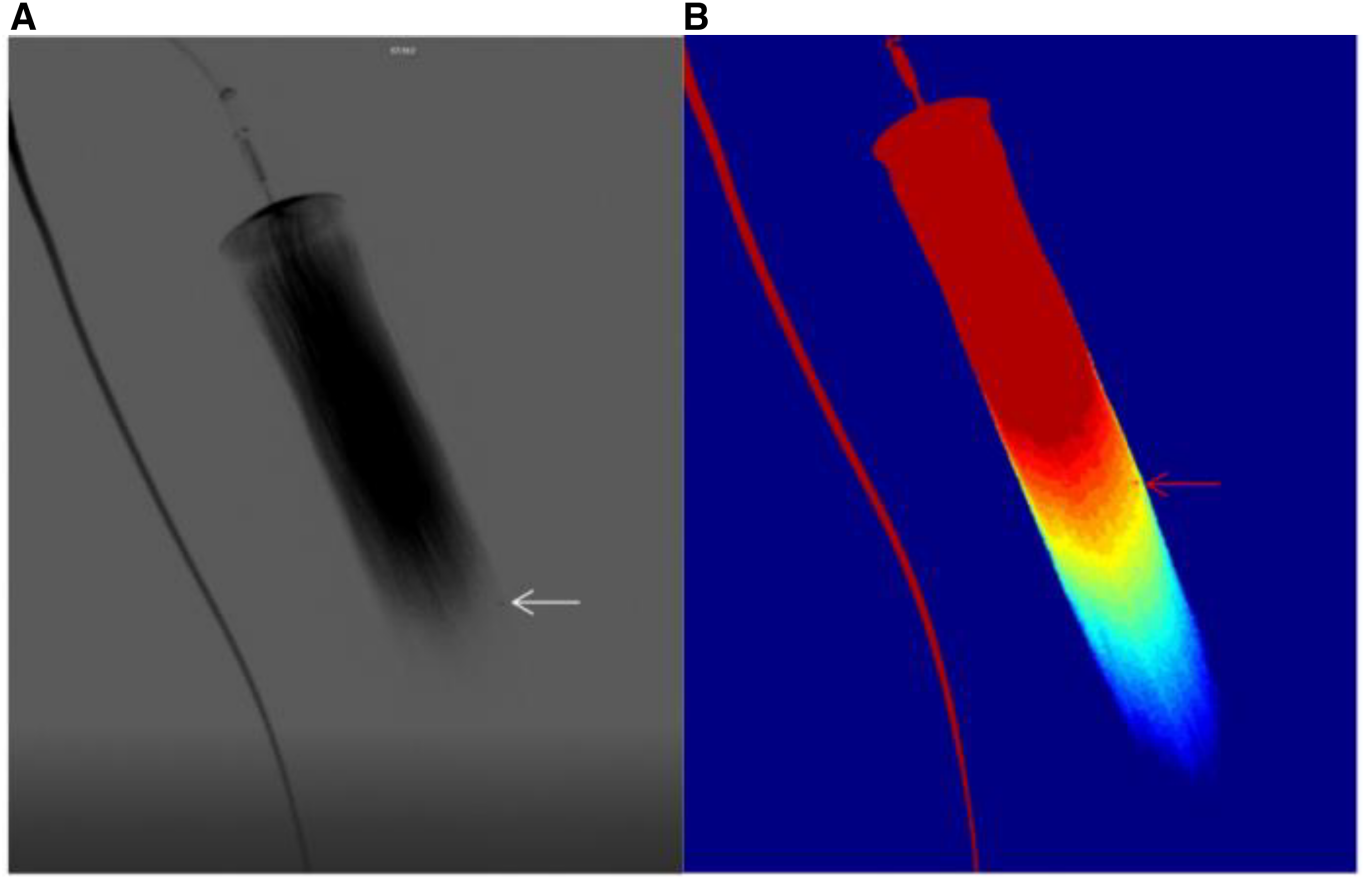
(**A**) shows the bleed shown by the DSA sequence, (**B**) shows the bleed shown by the time-of-arrival color parameter.

The ability of the 5 color parameters (arrival time, AUC, time to peak, transit time, contrast medium flow rate) to identify breakpoint of fibers was compared in 27 *in vitro* experiments (Figures 2, 3). The time-of-arrival color parameter imaging was the optimal color-coded parametric imaging to identify breakpoint, and this difference was statistically significant (*P *< 0.001).

### 3.2 The value of time-of-arrival color parametric imaging for diagnosis

Among the 27 *ex-vivo* experimental groups, 11 cases were diagnosed as definite breakpoint by DSA. 9 cases were diagnosed as probable breakpoint by DSA, in these nine cases, 7 cases were diagnosed as definite breakpoint and 2 cases were diagnosed as uncertain when combined with time-of-arrival color parametric imaging. 2 cases were diagnosed as uncertain by DSA, one case was diagnosed as definite breakpoint and another as probable breakpoint when combined with time-of-arrival color parametric imaging. Among the 11 cases that were unclear with DSA diagnosis (9 probable breakpoint cases and 2 uncertain cases), the combination of time-of-arrival color parametric imaging reinforce the diagnosis of breakpoint in 9 patients, and the difference was statistically significant (*P *< 0.01).

### 3.3 Clinical validation study

**Figure 4:**
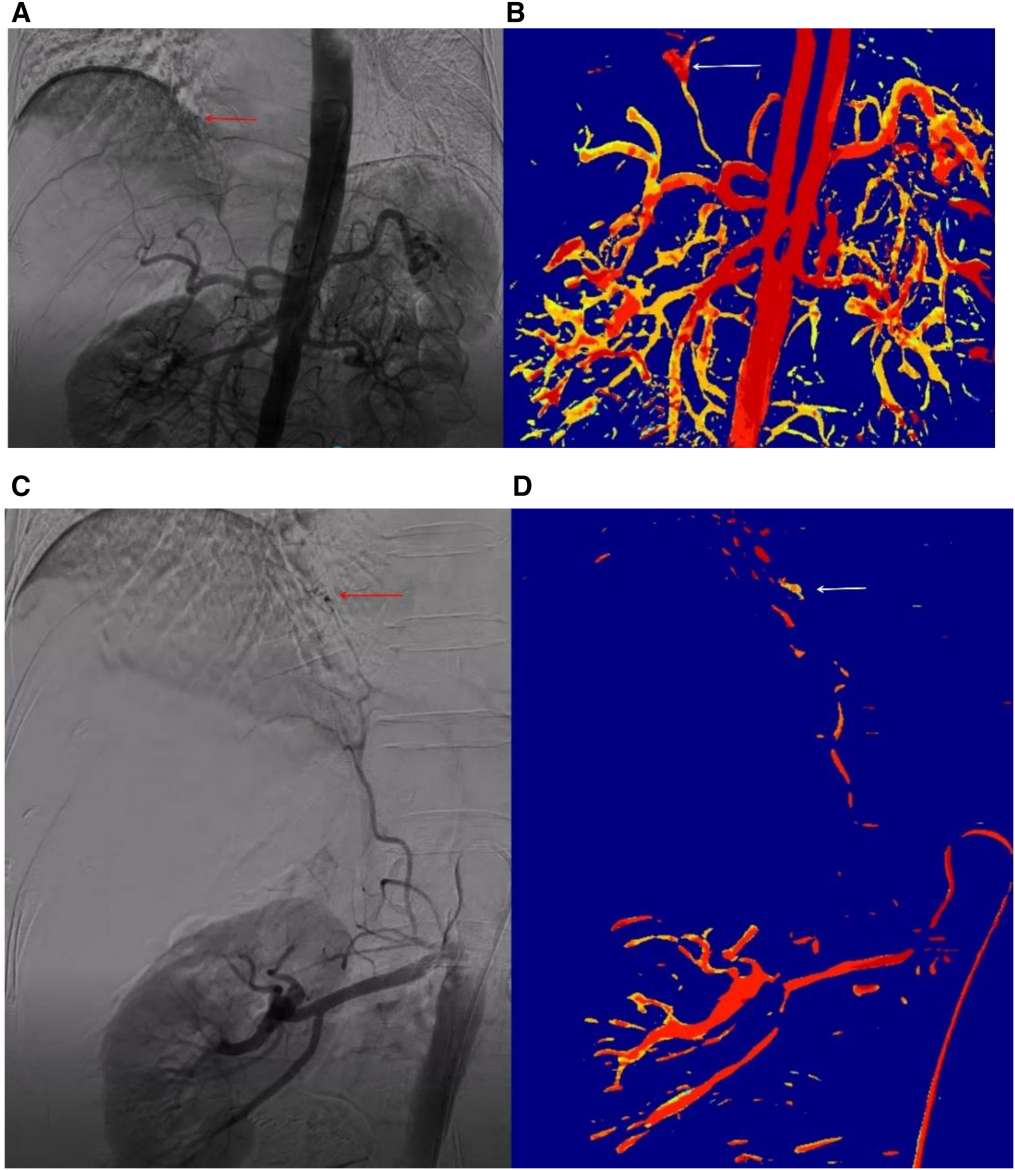
The DSA sequence and time-of-arrival color parameter of clinical bleeding case the patient was a 79-year-old female who experienced bleeding following a radiofrequency ablation procedure for hepatocellular carcinoma. No abnormalities were identified on hepatic arteriography, but suspicious hemorrhagic spots were visible on abdominal aorta angiography, albeit not clearly (**A**). Time-of-arrivial color parameter imaging of arrival time showed more obvious suspicious hemorrhagic spots (**B**). Renal arteriography showed the phrenic artery emanating from the renal artery and a hemorrhagic point (**C**). Time-of-arrival color parameter imaging also revealed the hemorrhagic point (**D**).

The time-of-arrival color-coded parametric imaging was applied in the clinic, with various colors representing arrival times from fast to slow. Red means the contrast agent arrives first, followed by yellow, and finally blue. The difference in the arrival time of the entire contrast agent is clearly displayed on one graph by the color difference. The time-of-arrival color parametric imaging (Figure 4) provides clear visualization of lesion location and morphology.

In the study, 135 patients were included, 101 of whom had definite bleeding and 34 of whom had no bleeding. The observations of DSA imaging and time-of-arrival color parametric imaging were performed blindly, with the process being the same as in the *ex-vivo* experiment.

Among the 101 patients with hemorrhage, according to the site of hemorrhage, 27 cases were liver hemorrhage, 22 cases were gastrointestinal hemorrhage, 25 cases were pulmonary hemorrhage, 15 cases were kidney hemorrhage, and 12 cases were pelvic hemorrhage. According to the causes of bleeding, 38 cases were caused by tumors, 10 were caused by trauma, 15 were caused by postpartum hemorrhage, 15 were caused by vascular malformation or aneurysm, 10 were caused by surgical operation, 5 were caused by radiotherapy, 4 were caused by portal hypertension, and 4 were caused by unknown cause.

Out of the 101 patients with clinically confirmed bleeding, 46 were diagnosed with bleeding by DSA. 48 were diagnosed with probable bleeding by DSA, and 28 (28/48) were diagnosed with confirmed bleeding using the combined time-of-arrival color coding imaging. Of the 7 cases diagnosed as uncertain by DSA, all were diagnosed as probable bleeding using the combined time-of-arrival color coding imaging. In the 55 patients who could not be diagnosed with bleeding by DSA (48 case with probable bleeding + 7 cases witn uncertain), the combined use of arrival time-of-arrival color coding imaging reinforce the diagnosis of bleeding in 35 cases (35/55), and the difference was statistically significant (*P *= 0.01, Table 1).

**Table 1:** 101 hemorrhage patients were diagnosed with DSA alone or DSA combining with time-of-arrival color parametric imaging.

Diagnosis/method	DSA	DSA + time-of-arrival	*P*-value
Bleeding	46	46 + 28	
Probably bleeding	48	20 + 7	
uncertain	7	0	0.01

Among the 34 patients with clinically confirmed no bleeding, 21 were diagnosed with no bleeding by DSA; 13 patients were diagnosed as probable no bleeding by DSA, but with the addition of time-of-arrival color coding imaging, they were confirmed as definitely non-bleeding. For the 13 patients diagnosed with probable no bleeding by DSA, the combined use of time-of-arrival color coding imaging was helpful in the diagnosis in all cases (13/13), and the difference was statistically significant (*P *= 0.03, Table 2).

**Table 2:** 34 patients with no bleeding were diagnosed with DSA alone or DSA combining with time-of-arrival color parametric imaging.

Diagnosis/method	DSA	DSA + time-of-arrival	*P*-value
No bleeding	21	21 + 13	
Probably no bleeding	13	0	0.03

## 4 Discussion

The study primarily used color-coded parametric imaging to transform 2D DSA raw data into color images based on parameters. The color-coded parametric imaging method combines gray-scale DSA sequence image information into a color image (13). This method provides the advantage of being able to quickly and accurately locate bleeding in clinical practice without increasing the operation time or radiation dose (14, 15). In this study, color-coded parametric imaging is obtained through image enhancement, vessel segmentation, time-intensity curve fitting, and image pseudocolor processing (16, 17). This study also compared the time-of-arrival color-parametric imaging with four other color-parametric imaging methods and found that the time-of-arrival color-parametric imaging had the highest identification rate for bleeding and was significantly higher than the other groups (*P *< 0.001). Through this study, we were able to identify the optimal color-parametric method for identifying hemorrhage among many color-parametric imaging methods, providing a theoretical basis for further clinical study.

In order to improve diagnosis and identify new lesions that may be missed by DSA, the impact of time-of-arrival color-coded parametric imaging on the diagnosis of breakpoint was analyzed. In the *ex-vivo* experiment, 11 cases that were unclear with DSA diagnosis (9 probable breakpoint cases and 2 uncertain cases), the combination of time-of-arrival color parametric imaging reinforce the diagnosis of breakpoint in 9 patients, and the difference was statistically significant (*P *< 0.01). These results suggest that when it is difficult to diagnose breakpoint with DSA alone, the combination of time-of-arrival color parametric imaging can provide additional clarification, making it a new and effective method for patients who have difficulty in diagnosing breakpoint through DSA (17). The time-of-arrival color parameter imaging method is particularly effective in reinforce dispaly of breakpoint.

To further confirm these results, DSA images of 135 patients (101 with confirmed bleeding and 34 with confirmed no bleeding) were collected. For patients whose bleeding could not be determined using DSA alone (55/101), the combination of time-of-arrival color parametric imaging was helpful for diagnosis, with a statistically significant difference (*P *< 0.01). These results indicate that for patients whose bleeding cannot be determined by DSA alone, the combination of time-of-arrival color parametric imaging can significantly aid in the diagnosis. For the 13 patients diagnosed with probable no bleeding by DSA, the combined use of time-of-arrival color coding imaging was helpful in the diagnosis in all cases (13/13), and the difference was statistically significant (*P *= 0.03) These results suggest that the combination of time-of-arrival color parametric imaging can significantly aid in the diagnosis of patients who cannot be confirmed as not having bleeding by DSA alone.

Based on the verification of these clinical cases, it can be concluded that time-of-arrival color parametric imaging is valuable in determining bleeding or no bleeding. The effectiveness of time-of-arrival color-coded parametric imaging in diagnosing bleeding was analyzed with the goal of improving accuracy and detecting new lesions that may have been missed by DSA. Among patients with confirmed bleeding, the combination of time-of-arrival color parametric imaging helped in the diagnosis of 35 cases out of 55 patients who were unable to determine bleeding by DSA alone. In these cases, the diagnosis of bleeding was improved in 30 cases, and new bleeding lesions were identified in 5 cases.

There were several limitations to this study. Firstly, the frame rate of DSA images used for parametric imaging was relatively low (generally 6fps in this study), resulting in low time resolution which may have hindered the identification of hemorrhagic sites in color-coded parametric imaging. Secondly, the current method of color coding analysis for DSA images involves converting DSA images into color images, which results in a loss of dynamic information (18). In future studies, we will explore the conversion of dynamic DSA sequences into dynamic color coding sequences. Additionally, the sample size for clinical verification was relatively small, and we will aim to increase the sample size in future analyses.

## 5 Conclusion

This study has the potential to enhance the efficiency and precision of analyzing active hemorrhage imaging data, as the use of color-coded parametric imaging enables quicker identification of crucial bleeding features. This could result in faster and more accurate diagnoses and ultimately, better patient outcomes. Among the five color parametric imaging methods, time-of-arrival color coding imaging was found to be the most effective in identifying bleeding.

## Data Availability

The original contributions presented in the study are included in the article/**Supplementary Material**, further inquiries can be directed to the corresponding authors.
