# Supplementary material for: Color-coded parametric imaging support display of vessel hemorrhage—an in vitro experiment and clinical validation study
Source: Front Cardiovasc Med. 2024 Jun 20;11:1387421. doi: 10.3389/fcvm.2024.1387421 (PMC11222601; doi:10.3389/fcvm.2024.1387421)
Supplement: Supplementary file 1 [file Datasheet1.doc]

**The ex-vivo experiment protocols of the active bleeding simulation study**

In this study, a German Braun polysulfone membrane, low flux dialyzer (model: LO PS 15) with a fiber inner diameter of 200 microns and a blood volume of 90 ml, and a membrane area of 1.5 m^2^ was used. The experimental procedure was divided into three steps: pretreatment of the dialyzer, connection of the device, and establishment of the in vitro simulation device.

The first step, the pretreatment of the dialyzer, involved opening a rectangular window of about 10cm × 5cm for easy cutting of fibers during the experiment to simulate bleeding (Figure 1).


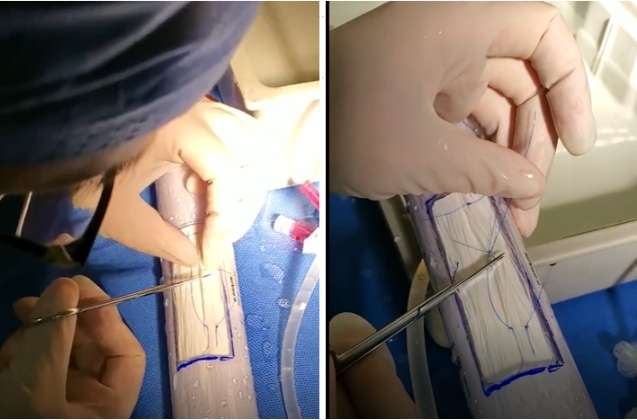


Figure 1: Simulation of bleeding by truncating fibers after dialyzer fenestration

The second step, the connection of the device, involved connecting the front end of the dialyzer to a peristaltic pump for control of water flow and placing it in a water tank. The water flow rate could be adjusted with the peristaltic pump.

The third step, the establishment of the in vitro simulation device, involved placing the dialyzer under a digital subtraction angiography machine (Philips angiography machine, FD20) and connecting a high-pressure syringe to the dialyzer through a needle to inject the contrast agent (iodixanol, Jiangsu Hengrui). This completed the model for the entire in vitro experiment.
