# Supplementary material for: Color-coded parametric imaging support display of vessel hemorrhage—an in vitro experiment and clinical validation study
Source: Front Cardiovasc Med. 2024 Jun 20;11:1387421. doi: 10.3389/fcvm.2024.1387421 (PMC11222601; doi:10.3389/fcvm.2024.1387421)
Supplement: Supplementary file 2 [file Datasheet2.doc]

**Orthogonal experimental design for DSA imaging parameter optimization**

During the ex-vivo simulation experiments, 8 parameters that may impact the performance of Digital Subtraction Angiography (DSA) images were selected. These parameters included the injection speed of contrast agent, the injection time of contrast agent, dose of X-ray, the number of DSA imaging frames, the injection pressure of contrast agent, the flow velocity (simulating blood flow rate), the number of broken fibers(simulating bleeding), and the distance of contrast agent injection from the injection point to the dialyzer.

Each parameter was set at 2-3 different levels, as follows: the injection speed of contrast agent(1ml/s, 2ml/s and 4ml/s), the injection time of contrast agent(1s, 2s and 4s), dose of X-ray (low, medium and high), the number of DSA imaging frames (3fps and 6fps), the injection pressure of contrast agent (200PSI, 400PSI and 600PSI), the flow rate(100ml/min and 200ml/min), the number of broken fibers (1, 2 and 4), and the injection distance of contrast agent (far: 50cm from the dialyzer, near: 0cm from the dialyzer). Parameters and levels were analyzed using SPSS software and 27 groups of experiments were conducted (as shown in Table 1). All statistical procedures were performed using SPSS software (IBM SPSS Statistics version: 23.0）.

The recognition of bleeding by the five color-coded parameter imaging methods along with arrival time + DSA in Table 2.

**Table1: The specific parameters of 27 experiments groups after the orthogonal experiment**

| **NO.** | **Speed**  **（ml/s）** | **Time**  **(s)** | **Dose** | **Frames**  **(fps)** | **Pressure**  **(PSI)** | **Flow velocity**  **（ml/min）** | **Number**  **(n)** | **Distance** |
| --- | --- | --- | --- | --- | --- | --- | --- | --- |
| 1 | 1 | 1 | low | 3 | 200 | 100 | 1 | near |
| 2 | 1 | 1 | low | 6 | 400 | 200 | 4 | near |
| 3 | 1 | 1 | low | 3 | 600 | 100 | 2 | far |
| 4 | 1 | 2 | high | 3 | 200 | 200 | 1 | far |
| 5 | 1 | 2 | high | 6 | 600 | 100 | 4 | near |
| 6 | 1 | 2 | high | 6 | 200 | 200 | 2 | far |
| 7 | 1 | 4 | medium | 3 | 600 | 200 | 1 | near |
| 8 | 1 | 4 | medium | 6 | 200 | 100 | 4 | far |
| 9 | 1 | 4 | medium | 3 | 400 | 100 | 2 | near |
| 10 | 2 | 1 | high | 3 | 600 | 200 | 4 | far |
| 11 | 2 | 1 | high | 6 | 200 | 200 | 2 | near |
| 12 | 2 | 1 | high | 3 | 400 | 100 | 1 | far |
| 13 | 2 | 2 | medium | 3 | 200 | 100 | 4 | near |
| 14 | 2 | 2 | medium | 6 | 400 | 200 | 2 | far |
| 15 | 2 | 2 | medium | 6 | 600 | 100 | 1 | near |
| 16 | 2 | 4 | low | 3 | 400 | 200 | 4 | near |
| 17 | 2 | 4 | low | 6 | 600 | 100 | 2 | far |
| 18 | 2 | 4 | low | 3 | 200 | 200 | 1 | far |
| 19 | 4 | 1 | medium | 3 | 400 | 100 | 2 | near |
| 20 | 4 | 1 | medium | 6 | 600 | 100 | 1 | far |
| 21 | 4 | 1 | medium | 6 | 200 | 200 | 4 | near |
| 22 | 4 | 2 | low | 3 | 600 | 200 | 2 | near |
| 23 | 4 | 2 | low | 6 | 200 | 200 | 1 | far |
| 24 | 4 | 2 | low | 3 | 400 | 100 | 4 | far |
| 25 | 4 | 4 | high | 3 | 200 | 100 | 2 | far |
| 26 | 4 | 4 | high | 6 | 400 | 200 | 1 | near |
| 27 | 4 | 4 | high | 6 | 600 | 100 | 4 | near |

Speed: speed of contrast agent injection. Time: the timing of contrast agent injection. Dose: dose of X-ray. low:349ugy/s, normal:697ugy/s,high:1350ugy/s. Frame rate: DSA imaging frame rate. Pressure: the injection pressure of contrast agent. Flow velocity: the speed of water circulation in vitro to simulate blood flow rate. Number: number of broken fibers. Distance: injection distance of contrast agent，far: 50cm from the dialyzer, near: 0cm from the dialyzer.

**Table 2：5 color-coded parametric imaging for identifying breakpoints**

| Group | DSA | arrival time | AUC | time to peak | transit time | contrast medium flow rate | DSA+arrival time |
| --- | --- | --- | --- | --- | --- | --- | --- |
| 1 | 1 | 1 | 1 | 1 | 1 | 1 | 1 |
| 2 | 1 | 1 | 1 | 1 | 1 | 1 | 1 |
| 3 | 1 | 1 | 1 | 1 | 1 | 1 | 1 |
| 4 | 4 | 4 | 1 | 1 | 1 | 1 | 5 |
| 5 | 1 | 1 | 1 | 1 | 1 | 1 | 1 |
| 6 | 4 | 4 | 1 | 1 | 1 | 1 | 5 |
| 7 | 5 | 5 | 1 | 1 | 1 | 1 | 5 |
| 8 | 4 | 1 | 1 | 1 | 1 | 1 | 4 |
| 9 | 5 | 4 | 1 | 1 | 1 | 1 | 5 |
| 10 | 3 | 1 | 1 | 1 | 1 | 1 | 4 |
| 11 | 4 | 3 | 1 | 1 | 1 | 1 | 4 |
| 12 | 1 | 1 | 1 | 1 | 1 | 1 | 3 |
| 13 | 5 | 4 | 1 | 1 | 1 | 1 | 5 |
| 14 | 5 | 4 | 1 | 1 | 1 | 1 | 5 |
| 15 | 4 | 5 | 1 | 1 | 1 | 1 | 5 |
| 16 | 5 | 4 | 1 | 1 | 1 | 1 | 5 |
| 17 | 3 | 3 | 1 | 1 | 1 | 1 | 5 |
| 18 | 5 | 5 | 1 | 1 | 1 | 1 | 5 |
| 19 | 5 | 4 | 1 | 1 | 1 | 1 | 5 |
| 20 | 5 | 5 | 1 | 1 | 1 | 1 | 5 |
| 21 | 4 | 5 | 1 | 1 | 1 | 1 | 5 |
| 22 | 5 | 4 | 1 | 1 | 1 | 1 | 5 |
| 23 | 4 | 5 | 1 | 1 | 1 | 3 | 5 |
| 24 | 4 | 4 | 1 | 1 | 3 | 1 | 5 |
| 25 | 4 | 5 | 4 | 4 | 1 | 4 | 5 |
| 26 | 5 | 5 | 3 | 1 | 1 | 1 | 5 |
| 27 | 5 | 4 | 4 | 4 | 3 | 1 | 5 |

1 point: definitely no breakpoint, 2 points: probably no breakpoint, 3 points: uncertain, 4 points: possible breakpoint, 5 points: definitely breakpoint.
